# Trends in Video Game Play through Childhood, Adolescence, and Emerging Adulthood

**DOI:** 10.1155/2013/301460

**Published:** 2013-03-20

**Authors:** Geoffrey L. Ream, Luther C. Elliott, Eloise Dunlap

**Affiliations:** ^1^School of Social Work, Adelphi University, P.O. Box 701, 1 South Avenue, Garden City, NY 11530, USA; ^2^Institute for Special Populations Research, National Development and Research Institutes, New York, NY, USA

## Abstract

This study explored the relationship between video gaming and age during childhood, adolescence, and emerging adulthood. It also examined whether “role incompatibility,” the theory that normative levels of substance use decrease through young adulthood as newly acquired adult roles create competing demands, generalizes to video gaming. Emerging adult video gamers (*n* = 702) recruited from video gaming contexts in New York City completed a computer-assisted personal interview and life-history calendar. All four video gaming indicators—days/week played, school/work day play, nonschool/work day play, and problem play—had significant curvilinear relationships with age. The “shape” of video gaming's relationship with age is, therefore, similar to that of substance use, but video gaming appears to peak earlier in life than substance use, that is, in late adolescence rather than emerging adulthood. Of the four video gaming indicators, role incompatibility only significantly affected school/work day play, the dimension with the clearest potential to interfere with life obligations.

## 1. Introduction

### 1.1. Literature Review

Video games are an indelible part of the modern American early life course. A 2008 Pew Research Center survey found that 99% of males and 94% of females ages 12–17 play video games [1]. Video gaming begins in early childhood [2] and continues through adulthood [3]. Video games are a modality for instruction [4] and clinical intervention [5]. They facilitate cognitive development [6]. They provide experiences of freedom and competence [7], opportunities to socialize, a sense of mastery, a medium for identity development, and—not least—fun [8]. Video games focus attention in ways that are palliative for ADHD [9, 10] and mood disorders [11]. A “downside” is the development among between 4.9% and 9% of video gamers of problem video game play (PVGP), an addiction-like pattern of feeling out of control of time spent playing, neglecting normal responsibilities in order to play, and so forth [12–18]. PVGP is distinct from merely liking video games or spending a lot of time playing them [19–21]. The validity of PVGP is not only supported by survey and laboratory research. Players recognize elements of PVGP within their own experiences [22] and there are clinical screening instruments for it [23]. PVGP is among the “behavioral addictions” correlated with substance dependence [24–26].

Because substance and behavioral addictions have similar biological mechanisms [27–30], it may be possible to generalize theories about life trajectories of substance use and dependence to those of video game play and problem play. According to our current understanding [31], adolescence and emerging adulthood are critical periods for emergence of substance use and dependence. Adolescents, through typical adolescent experimentation with substances, discover substances' usefulness for regulating negative emotions and then use them specifically for this purpose in emerging adulthood. This is a potential problem for public health reasons, as “self-medication” is connected with addiction. It is also a source of concern for developmental reasons, as growing through disequilibrating experiences is necessary for identity development [32], and self-medicating “the pain of growing up” instead of constructively confronting it may impede progress through developmental tasks [33, 34]. It is reasonable to suspect that video games have the potential to play a similar role to substances', as youth discover video games' potential for regulating negative emotions early on. A survey of middle school students found that 62% of boys and 44% of girls used video games to help them relax, 45% of boys and 29% of girls used them to cope with anger, and smaller numbers used them to forget problems and cope with loneliness [8]; some players intentionally use video games to escape real-life problems [20, 35].

For the average youth, substance use decreases through emerging and young adulthood as, according to role incompatibility theory, competing demands of school, work, and relationships make previous substance use levels untenable [36–38]. However, some substance users, perhaps having developed addictive disorders, continue high levels of use and disengage from developmental tasks [31], with predictable consequences for both health and psychosocial development. There is already reason to believe that consistent video game play has long-term health consequences, with recent findings of lower mental and physical health among adult video gamers [39]. The present study explored the potential for role incompatibility effects on video gaming behavior.

### 1.2. Research Questions

If video gaming, or at least certain patterns of video gaming, fills a similar role in the life course to that of substance use, then average levels of duration/frequency of video gaming and PVGP should follow the same trends across the early life course as substance use, that is, rising through childhood and adolescence to an inflection point and then falling through emerging adulthood as competing demands of adult roles make previous levels of play untenable [31, 33, 40]. Analyses for the present study, therefore, tested the hypothesis that relationships between age and four different video gaming indicators—days/week of play, school/work day play, non-school/work day play, and problem play—were significantly curvilinear (i.e., with negative coefficients for quadratic terms indicating a downward inflection). Also consistent with the idea that video gaming responds similarly to life-course pressures as substance use, our analyses tested the hypothesis that the curvilinearity of the relationships between at least some video gaming indicators and age was statistically explained by role incompatibility [38, 40], operationalized using indicators of acquisition of adult roles of full-time work, higher education, and independent living. We expected the pattern of significant and nonsignificant coefficients for age and adult role indicators not to change even after controlling for known covariates of video gaming, including sugar and caffeine consumption [19, 41–44], personality [45], gender [46, 47], and race [48, 49].

## 2. Methods

### 2.1. Participants

Participants were emerging/young adults ages 18–29 recruited in and around 52 different video game stores, arcades, internet/cyber cafes, game-themed convention booths, and retail stores with large video game departments in New York City. Time and day of data collection were varied to obtain a diverse sample. Quota sampling was used to obtain at least 10 participants for every “cell” that would be created by cross-tabulating gender, race, and illicit substance user status, with some participants screened out if they were not needed to meet a quota. Initial contact was made with 1090 potential participants. Of these, 150 were ineligible or screened out, and 238 declined to participate. If participants were interested but could not complete the interview at that time, interviewers set appointments. Ten cases were invalid because of incomplete responses. Our total valid n was 692, for a response rate of 692/940 = 74%. 

### 2.2. Procedure

Field interviewers took participants to a mutually agreeable public location for the interview, often a park, fast-food restaurant, or coffee shop. Measures included a computer-assisted personal interview (CAPI) for time-invariant indicators, including demographics and personality variables. Time-varying variables including life-course indicators, caffeine and sugar consumption, and video game playing were measured via a quantitative life-history calendar (LHC; [50–52]). The instrument itself was a computerized spreadsheet with a column for each year of life ages 6–29 and a row for each variable, so that participants answered each LHC question once for every year of life ages 6–present. This turned out to be not as tedious or fatiguing as we thought it would be before we pilot-tested it during measurement development; participants appeared to enjoy talking about their video gaming histories and watching the calendar grid fill up in front of them. Participants were compensated $30 plus any refreshments interviewers bought for them with a $5 per interviewee budget. The protocol was approved by the investigators' institutional review boards.

### 2.3. Measures

#### 2.3.1. Frequency/Duration Video Gaming and Problem Play

The LHC included three questions about duration/frequency of video gaming. Days/week of play was measured by “In a typical week, on how many days did you play games?” Separate questions were included for school/work day play: “On weekdays (or days that you had to go to school or work), how many hours per day did you play video games?” and nonschool/work day play: “On weekends (or days that you did not also have to go to school or work), how many hours per day did you play video games?” because the former seemed more likely to interfere with life obligations [53]. Responses were open-ended; the handful of responses that were not whole numbers were rounded to the nearest whole number for analyses. The LHC question for problem play (PVGP) was “Think about the problem video game playing criteria from the computerized interview—what was your degree of problem video game playing?” according to the scale of 0 = none, 1 = slight, 2 = moderate, 3 = high, and 4 = extreme. Although these single-item measures are probably more prone to random error than a multiple-item construct would have been, they were the only means by which all of the needed data could be collected in one sitting. Interviewers attempted to maximize the validity of these single-item responses in the LHC by referring respondents back to the standardized measures for present-day PVGP (not used in these longitudinal analyses) which they had already completed in the CAPI.

#### 2.3.2. Sugar/Junk Food Consumption

This was the average of responses to one question about sugar drinks and another about high-sugar food, each on a 6-point Likert scale ranging from 0 = “never or less than once a week” to 5 = “exclusively—it was almost all I [ate/drank].”

#### 2.3.3. Caffeine

Caffeinated drinks/day was the number of caffeinated drinks or caffeine pills the participant reported on days of caffeine use. Degree of problem use was a single-item measure with the same 5-point Likert scale as problem video game play. Zeroes were imputed for nonusers.

#### 2.3.4. Adult Role Indicators

For each year, each of these factors was coded 1 if they applied to the participant for more than half of the year and 0 if not. They included whether the participant attended a 2-year or 4-year school, whether they held a full-time job, whether they lived in student housing, and whether they lived away from home (i.e., biological, adoptive, or foster family).

#### 2.3.5. Personality

Personality measures were adapted from the National Longitudinal Survey of Adolescent Health (“Add Health” [54]). They included sensation-seeking, 13 items, Cronbach's *α* = .64, shyness, seven items, *α* = .73, and sociability, three items, *α* = .69. These modest *α*'s may have made these variables less competitive for variance in multivariate models.

#### 2.3.6. Demographics

These included race, gender, and age at the time of the interview.

### 2.4. Analysis Plan

 Our analyses built upon an existing example of multilevel modeling (MLM) for LHC data [52], treating year-level observations as nested within individuals. Because few participants were older than 26 at the time of the interview, data for those ages were sparse and subject to apparent selection biases. Since this study was not concerned with trends beyond the end of emerging adulthood anyway, we used only data for ages ≤ 26. Each inferential model was a multivariate multilevel model conducted in Mplus 6.0 with days/week of play, school/work day play, and nonschool/work day play as count dependent variables and problem play as a continuous dependent variable. All continuous variables were grand-mean centered. Because model fit and percentage of variance explained statistics are not available in Mplus for count dependent variables, model fit was approximated using alternative models in which days/week of play, school/work day play, and nonschool/work day play were specified as continuous dependent variables. Standardized coefficients (STDYX in Mplus) are reported throughout. Although we ran a total of four models in order to distinguish the role incompatibility effect, we summarize relevant results from the first three in text and, in the interest of space, only describe the final model in a table.

## 3. Results

Participants were 22% white, 24% African-American/Black, 20% Latino, 20% Asian, and 14% other/mixed. About two-thirds (66%) were male. Mean age at the time of the interview was 21.2, SD = 3.1. Most (58%) had spent at least half a year as a 2-year or 4-year college student, and 42% reported at least half a year of full-time work. At least half a year of living in student housing was reported by 20%. Only 1% of participants had never lived away from home at the time of the interview; 5% were living away from home at age 17, 14% at 18, 49% at 19, 63% at 20, 76% at 21, 84% at 22, 88% at 23, and above 90% at later ages. Personality variables were roughly normally distributed and their means were close to their scales' middle ranges (all 1–5): For shyness, *M* = 2.3, SD = 0.7; for sensation-seeking, *M* = 3.3, SD = 0.6; for sociability, *M* = 3.5, SD = 0.8.

**Figure 1:**
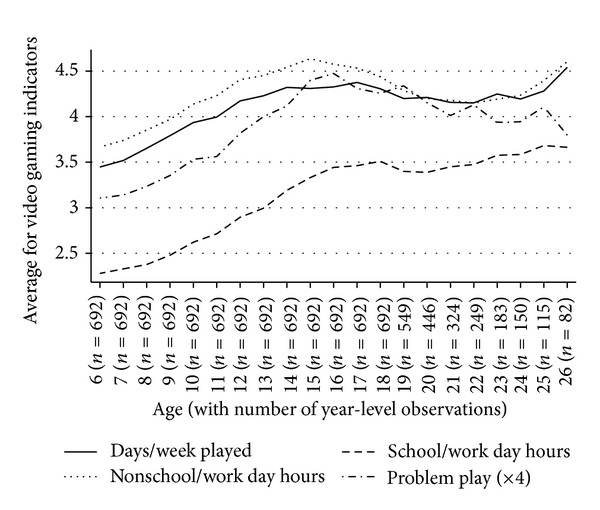
Trends in video gaming variables through the early life course. All curves were significantly quadratic in analyses that did not include control variables.

Figure 1 describes trends in video gaming variables over childhood, adolescence, and emerging adulthood. Problem play scores were multiplied by four for display in Figure 1 so that they would fit into the same *y*-axis range as the frequency/duration indicators. All four basic relationships depicted in Figure 1 were significantly curvilinear, according to results of multivariate multilevel models (table not shown; CFI and TLI > .999, RMSEA and SRMR < .001, within-level *R* ^2^'s between .02 and .09 and corresponding *P*'s ≤ .001) predicting all four video gaming indicators from linear and quadratic terms for age and controlling only for age at time of interview. In spite of the second inflection point around ages 21-22 that appears to emerge in Figure 1, no cubic terms were significant in these analyses and would have only been supported by data for less than half of participants if they had been significant. All four quadratic terms remained significant in a second analysis that included controls for caffeine and sugar consumption, race, gender, and personality, indicating that the curvilinear shape of the relationship between video gaming indicators and age was not affected by their entry into the model. In a third analysis in which these controls were removed and adult role indicators of higher education involvement, full-time work, living away from home, and living in student housing were introduced, the quadratic term for age as a predictor of school/work day play became nonsignificant, and all other quadratic terms remained significant, indicating that there was some shared variance in school/work day play only between the curvilinearity of its relationship with age and the life-course indicators. Additional findings from that model were that living away from home had a negative partial relationship with days/week of play, higher education involvement had a negative partial relationship with nonschool/work day play, and both variables had negative partial relationships with school/work day play.

Table 1 describes the fourth and final model, which includes adult role variables and controls. The quadratic term for age as a predictor of school/work day play remained nonsignificant and the only remaining significant partial relationships with adult role indicators were with school/work day play. Taken together with results from the reduced models, the general finding is that the life course indicators as a set explained variance in school/work day play that would otherwise have been attributable to the quadratic term for age. These life-course variables were (predictably) mostly constant throughout childhood and early adolescence; they could only have covaried with and explained variance in dependent variables through late adolescence and emerging adulthood. Therefore, it could reasonably be concluded that these indicators of transitions to adult roles explained the downward inflection in the relationship between school/work day play and age in late adolescence and emerging adulthood. This effect did not occur for any other video gaming indicator—although living away from home had a negative partial relationship with days/week of play, the quadratic term for age was still significant after adding life-course variables to the model.

Other findings in the final model were that caffeine use reliably predicted video game play, problem caffeine use predicted PVGP, and sugar/junk food consumption was associated with all indicators of video game play and PVGP. Black, Latino, and other/multiracial respondents played video games more often and longer than whites, and Latino and Asian respondents reported more PVGP than white respondents. Personality was generally unrelated to video gaming except for a significant association between shyness and PVGP; these tests may have been underpowered due to the marginal reliability of the measures. 

## 4. Discussion

 Results confirmed hypotheses that all four video gaming indicators studied—days/week of play, school/work day play, non-school/work day play, and problem play (PVGP)—had curvilinear relationships with age, rising through childhood and adolescence to a peak and then leveling off or decreasing in emerging adulthood. Other than that the inflection point was apparently earlier in the life course for video games than it is for substances (a common sense explanation for which would be that adolescents have easier access to video games than to substances) these trends were congruent with those which earlier research found between age and substance use [31, 40]. We also found the hypothesized “role incompatibility” [37, 38] effect, but only for school/work day play. Although the hypothesis which was only confirmed for one indicator would seem to weaken the case of role incompatibility with video gaming, school/work day play is the dimension with the clearest potential to interfere with life obligations, and interference with life obligations is an important distinguisher between benign and problematic patterns of video game play [53]. The findings could, therefore, be interpreted as indicating that, although video gaming generally levels off or decreases in emerging adulthood, this leveling off is only attributable to role incompatibility for video gaming that interferes with other life responsibilities. The evidence for this interpretation would, of course, be stronger if a similar finding had also emerged for problem play. 

This study's primary strength was its developmental/life-course perspective. Although video gaming studies usually focus on children and adolescents [55, 56], they do not often apply a developmental perspective. Another strength was its face-to-face interviewing methodology, which held participants to task so that they finished the survey and did not contrive responses. The LHC format was not only enjoyable for participants but allowed us to operationalize age as a continuous independent variable. Our study, like other LHC studies [50, 51], is limited in that data were retrospective, not truly longitudinal. Our participants often gave single responses for several-year increments, essentially imputing an average across several years in place of the randomly varying responses of a true longitudinal measure. Since one of MLM's most important features is adjustment for inflated likelihood of type I error due to nonindependence of observations within level, our use of MLM [52] gives us the best chance of valid results in spite of this limitation, assuming these averages across years were not actually biased. However, the conceptual limitation remains that there were fewer distinct time points in the minds of participants than were represented in analyses. This study was also limited, as aforementioned, by the necessity of reliance on single-item indicators. Findings are also not necessarily generalizable beyond the population of emerging adults who frequent NYC video gaming contexts.

Although conclusions about development based on retrospective data are admittedly tentative, our findings may be used to support a case for gathering true longitudinal data, perhaps through including additional questions about video games and other media use in large-scale survey studies of adolescents like Monitoring the Future [57] or Add Health [54]. Video gaming is, after all, endemic to the early life course, and these and other findings suggest that it may have long-term influences on health and development.

## Figures and Tables

**Table 1:** Results of multilevel model predicting video gaming habits from study variables.

	Days/week played	School/work day hours/day played	Nonschool/work day hours/day played	Problem play (PVGP)
Year-level variables:				

Age (linear)	1.86***	1.51***	1.37**	0.42***
Age (quadratic)	−1.37***	−0.59^a^	−0.99*	−0.29*
Caffeinated drinks/day	0.27**	0.29**	0.36**	−0.06
Caffeine problem use	−0.06	0.02	−0.04	0.18***
Soda/junk food consumption	0.65***	0.32***	0.68***	0.18***
Attending 2yr or 4yr college	0.02	−0.14*	−0.08	0.01
Holding full-time job	0.04	−0.15*	−0.17	0.02
Living in student housing	−0.08	0.003	−0.05	0.01
Living away from home	−0.20*	−0.13	−0.04	−0.03

Participant-level variables:				

Race: Black	0.27*	0.29^+^	0.41**	−0.02
Race: Latino	0.65***	0.64***	0.64***	0.11*
Race: Asian	0.11	0.01	−0.02	0.11*
Race: Other/multiracial	0.41***	0.41**	0.50***	−0.03
Gender: Female	−0.71***	−0.59***	−0.61***	−0.17***
Personality: Sensation-seeking	0.18	0.26	0.21	0.04
Personality: Shyness	0.07	0.09	0.12	0.09*
Personality: Sociability	0.14	0.19	0.02	0.04
Age at time of interview	−0.03	−0.20	−0.15	−0.05

Model characteristics:				

Intraclass correlation	0.48	0.45	0.55	0.67
*R*-squared within	0.12 ***	0.17 ***	0.13 ***	0.09***
*R*-squared between	0.14 ***	0.08 ***	0.08 ***	0.07***

CFI and TLI > .999, RMSEA and SRMR < .001. Coefficients are standardized. Because model fit statistics, intraclass correlations, and *R*-squared values are not available in MPlus for models including count dependent variables, these model fit statistics and the italicized *R*-squared values are taken from an alternative model (also run using MPlus) in which days played and hours/day played were specified as continuous dependent variables. ^*+*^ *P* < .10, *P < .05, **P < .01, and ***P < .001. ^a^This coefficient was significant in models that did not include life-course indicators.
